# Effects of birth-rearing type on weaning weights in meat sheep are systematically associated with differences in mean performance among flocks

**DOI:** 10.1186/s12711-015-0136-2

**Published:** 2015-07-03

**Authors:** David R. Notter, Daniel J. Brown

**Affiliations:** Department of Animal and Poultry Sciences, Virginia Tech, Blacksburg, VA 24061 USA; Animal Genetics and Breeding Unit, University of New England, Armidale, NSW 2351 Australia

## Abstract

**Background:**

Adjustment of body weights for systematic environmental effects such as dam age and litter size is essential for accurate prediction of breeding values in meat sheep and often accomplished by pre-adjusting records using simple multiplicative adjustment factors, which are derived as ratios of least-squares means of weights of lambs in target and reference classes. However, increasing use of multibreed genetic evaluations that incorporate data from both purebred and commercial flocks has generated concerns regarding the ability of simple additive or multiplicative adjustment factors to properly correct for environmental effects in flocks that differ widely in mean performance. Thus, consistency of adjustment factors across flocks and systematic effects of the level of flock performance on these factors were evaluated using data from the US National Sheep Improvement Program.

**Methods:**

We used birth and weaning weights of lambs from 29 flocks that had at least 500 records per flock and represented several terminal-sire sheep breeds. Effects of lamb sex, dam age class and litter size on birth weights, and of dam age class and combined effects of type of birth and rearing on weaning weights were evaluated. Interactions between these effects and flock were assessed. Bias associated with different adjustment protocols was evaluated for high- and low-performance flocks.

**Results:**

Effects of litter size and differences between yearling and adult dams varied (*P* < 0.001) among flocks. For weaning weights, additive adjustment factors were not associated with the level of flock performance, but multiplicative adjustment factors were strongly and inversely related to flock means for weaning weights (W). Flock-specific adjustment factors (F = αW^β^) reduced bias in adjusted weaning weights associated with differences in flock performance. By contrast, simple multiplicative adjustment factors were appropriate to adjust birth weights.

**Conclusions:**

Differences in weaning weights among single, twin, and triplet lambs were inversely related to the level of flock performance. Use of simple multiplicative adjustment factors led to adjustment bias when applied across flocks with large differences in mean performance. This bias was reduced by using additive adjustment factors or multiplicative factors that were derived as simple exponential functions of flock means for weaning weight.

## Background

Adjustment of body weight records for systematic environmental effects such as age of the dam and number of lambs born and reared in a litter is essential for accurate prediction of breeding values (BV) in meat sheep. In most cases, these adjustments are implemented by either including these effects in the statistical model used for BV prediction or applying predetermined adjustment factors to phenotypic records before BV prediction. The former approach allows environmental effects to be estimated simultaneously with genetic effects in the statistical model and, for most models used in BV prediction, it results in additive adjustments for the effects of these variables. By contrast, preadjustment of records before statistical analysis often involves the use of multiplicative adjustment factors that are commonly considered to be more robust for modeling environmental effects across flocks that differ in mean performance. These multiplicative factors are normally derived from records of the animals to be evaluated as ratios of least-squares means for weights of lambs in different effect classes and they are periodically updated as additional data accumulate. Use of external adjustment factors also allows the use of more complex nonlinear adjustments. For example, Notter et al. [1] derived an *ad hoc* nonlinear predictor of continuous effects of dam age on lamb birth and weaning weights that was more effective than a simple linear and quadratic polynomial predictor but could not be easily integrated into standard mixed linear models.

If flocks and effect classes are large and if the number of management groups present within each flock and year is small, the adjustment strategy used is unlikely to have important effects on the results. Effects of lamb sex, dam age, and birth-rearing type on body weights can be fitted in the statistical model, and interactions of these effects with each other and with other fixed effects such as flock and year can be tested and included in the model as needed. However, if small flocks, infrequent class effects, and significant interactions are present in the data, more attention is required to develop adjustment protocols. Including interactions as fixed effects can lead to spurious results if subclass numbers are small, and the resulting least-squares constants become more liable to sampling errors. Then, fitting interaction effects as random effects becomes essential, to allow the effects’ constants that are derived from small subclasses to be regressed towards the population mean. In most cases, pre-adjustment of records becomes increasingly attractive as the complexity of the data and the incidence of small subclasses increase, which allows greater control over the factors being applied and avoids producing biologically unreasonable results that are associated with sampling errors or nonrandom assignment of individuals to management groups.

Use of multibreed genetic evaluations is increasing, with the goal of providing industry-wide BV predictions for animals in dual-purpose, maternal, and terminal-sire breeds in both seedstock and commercial flocks [2]. However, multibreed datasets and datasets that include both seedstock and commercial flocks may also exhibit greater diversity in mean performance across flocks, which raises questions regarding the ability of simple additive or multiplicative adjustment factors to properly correct for environmental effects across flocks. We found that the use of a common set of multiplicative factors to adjust lamb weaning weights for effects of birth-rearing class in a set of meat breeds including Suffolk, Hampshire, Dorset, Shropshire, and multibreed composite flocks in the US National Sheep Improvement Program (NSIP) appeared to result in higher mean EBV for single-born lambs in flocks that emphasized forage-based production and had lower lamb pre-weaning gains compared to flocks that were offered higher levels of concentrate feeding. This apparent bias was a concern in terms of accuracies of BV predictions and as a source of bias against increasing prolificacy. Thus, among the flocks analyzed, we investigated the variation in factors that are necessary to adjust lamb birth and 60-day weaning weights for effects of birth-rearing type and dam age class and associations of resulting flock-specific factors with flock means for body weights.

## Methods

Analyses in this study used only records from relatively large flocks and were designed to: (1) determine the effects on birth and weaning weights of flock × dam age class and flock × birth (for birth weight) or birth-rearing type (for weaning weight) interactions; (2) derive additive and multiplicative adjustment factors for these effects for individual flocks; (3) investigate relationships between flock-specific factors and flock characteristics; (4) assess the need for, and develop strategies to, implement flock-specific adjustment factors; and (5) quantify effects of alternative adjustment procedures on adjusted weights.

### Data

Records were available for birth and (or) weaning weights of 36 949 lambs from 123 flocks that were evaluated in 1988 through 2011; these flocks participated in the NSIP Terminal Sire Breed evaluation program [3] and were maintained in the Australian Sheep Genetics LAMBPLAN database [4]. In order to estimate flock-specific adjustment factors with reasonable accuracy and facilitate the detection of relationships between resulting adjustment factors and flock characteristics, only flocks with at least 500 records for birth or weaning weights were used, yielding initial sets of 23 and 28 flocks, respectively. Twenty nine different flocks were represented in the datasets and included 20 Suffolk, five Dorset, one Hampshire, one Shropshire, and two crossbred composite flocks. Frequency tables were used to assess the distribution of observations among dam age classes (1, 2, 3 to 6, or > 6 years of age at lambing, corresponding to dam ages of 8 to 17, 18 to 29, 30 to 66, and > 66 months, respectively and hereafter referred to as yearling, 2-year-old, adult, and aged dams, respectively); types of birth-rearing (TBR; restricted to single, twin, and triplet births for birth weight and to TBR = 11, 22, and 33 for weaning weights); and birth seasons (winter = December through February; spring = March through May; summer = June through August; autumn = September through November). Preliminary analyses confirmed that dam age effects within the adult dam age class were small and not significant. Flock × season subclasses with fewer than 50 observations were deleted, which resulted in the exclusion of all summer-born lambs. The incidence of autumn lambing was higher for Dorset flocks and affected variation in dam age at lambing within dam age classes. To reduce variation among flocks in mean dam age at lambing within yearling and 2-year-old dam age classes, records in these classes were restricted to ewes that lambed at 11 to 14.5 and 21.5 to 26.5 months of age, respectively.

A core dataset was created for each trait (Table 1) by requiring at least 50 observations per flock for each dam age class and birth-rearing type. Core datasets were used to compare: (i) 2-year-old and adult dams; (ii) birth weights of single, twin, and triplet lambs; and (iii) weaning weights of single-born, single-reared and twin-born, twin-reared lambs (i.e., TBR = 11 and 22). For less-numerous yearling and aged dams, satisfying the requisite of 50 observations in each flock for each effect class resulted in smaller datasets with fewer flocks (Table 1). These datasets included 2-year-old, adult, and either yearling or aged dams and only lambs born as single births and twins for the birth weight analysis or with TBR = 11 and 22 for weaning weights.

**Table 1:** Distribution of observations among dam age classes and among birth (TB) or birth-rearing (TBR) types for birth and weaning weights

Weight	Data set	Number of flocks	Number of observations	Effect	Effect class	Number of observations per class	Number of observations per flock		
							Median	Minimum	Maximum
Birth	Core	23	24 621	TB	1	4640	162	60	905
					2	15 718	556	269	2034
					3	4263	159	50	490
				Dam age	2	7140	242	122	889
					3 to 6	17 481	636	298	2339
				Lamb sex	Females	12 165	429	218	1591
					Males	12 456	461	221	1637
	Yearling dams^a^	15	18 211	Dam age	1	2952	152	59	687
					3 to 6	10 465	535	183	2061
	Aged dams^b^	17	23 430	Dam age	>6	2328	98	54	438
					3 to 6	15 134	757	302	2339
Weaning	Core	28	19 476	TBR	11	5002	142	45	877
					22	14 474	410	227	1705
				Dam age	2	6166	173	89	695
					3 to 6	13 310	367	174	1581
	TBR = 33^c^	20	17 438	TBR	33	1577	58	30	202
					22	11 775	503	227	1705
	Yearling dams^a^	15	14 869	Dam age	1	2545	140	50	548
					3 to 6	8265	431	174	1581
	Aged dams^b^	10	11 835	Dam age	>6	1230	97	53	309
					3 to 6	7444	589	286	1581

^a^Includes flocks from the core dataset that have at least 50 records on yearling dams. These datasets were restricted to only single and twin lambs but included lambs with 2-year-old dams. Numbers are shown only for the target and reference dam age classes

^b^Includes flocks from the core dataset that also have at least 50 records on > 6-year-old dams

^c^Includes flocks from the core dataset that also have at least 30 records for lambs with TBR = 33. This dataset was restricted to only 2-year-old and adult dams but included lambs with TBR = 11. Numbers are shown only for the target and reference birth-rearing types

Triplet lambs were relatively frequent in these data (17 % of lambs born in the core birth weight data) and triplet rearing was common (47 % of surviving triplet lambs). Adjustment factors for weaning weights for lambs with TBR = 33 were therefore derived from a dataset that was restricted to 2-year-old and adult dams (Table 1). The minimum number of observations for TBR = 33 was reduced to 30 per flock to allow the use of data from more flocks. Datasets including lambs with TBR = 32 or TBR = 21 and with more than 30 lambs per flock in these birth-rearing types were also created and were similar in size to the TBR = 33 dataset in Table 1. Lambs with TBR = 12 or TBR = 31 were rare, and these records were excluded from the data.

### Statistical methods

Each dataset in Table 1 was initially analyzed with a mixed linear model:

$$ \mathbf{Y}=\mathbf{X}\boldsymbol{\upbeta } +\mathbf{Z}\mathbf{u}+\boldsymbol{\upvarepsilon}, $$

where **Y** is a vector of birth or weaning weights; **β** and **u** are vectors of fixed and random effects, respectively, with corresponding design matrices **X** and **Z**; and **ε** is a vector of residual effects. Weaning weights were adjusted to a standard age of 60 days before analysis using LAMBPLAN protocols [5]. The main purpose of the analysis was to assess the magnitude of the effects of flock × dam age class and flock × birth (or birth-rearing) type interactions, and the model included fixed effects of flock, birth year, dam age class, birth-rearing type, and interactions of dam age class × birth-rearing type, flock × dam age class, and flock × birth-rearing type. Random effects included management group effects (described in detail below); flock × dam age class × year and flock × birth-rearing class × year interaction effects; and residual errors. The birth weight analysis also included fixed effects of lamb sex and flock × lamb sex interaction and random flock × lamb sex × year interaction. Since effects of lamb sex × birth-rearing class and lamb sex × dam age class interactions, which were tested in preliminary analyses, were found to be non-significant, they were excluded from the final birth weight analyses. Management groups for birth weights were defined by the flock, year, and season of birth (defined using rolling 35-day birth date windows). Management groups for weaning weights included the flock, year, season of birth, weaning date, sex of the lamb (ewe, ram, or wether (i.e. castrated male sheep)), and a producer-defined management code in accord with LAMBPLAN protocols [6]. Effects of flock × dam age class and flock × birth-rearing class interactions were tested using the effects of flock × dam age class × year and flock × birth-rearing class × year interactions, respectively, to account for variation in flock-specific effects among years and assess the repeatability of flock effects on dam age class and birth-rearing type among years.

For each flock, mean effects of flock × dam age class and flock × birth-rearing type interactions from this model were used to derive additive and multiplicative adjustment factors as differences from, and ratios to, respectively, least-squares means for reference classes (i.e., lambs from adult dams, and lambs born and reared as twins). Single lambs have generally been used as the reference class in sheep genetic evaluations, but, for this study, we preferred to use twin lambs which were much more frequent. These factors were then plotted against flock means for the reference classes, and associations were determined after weighting the factors for each flock by the number of observations in the smallest class used to calculate the factor (Table 1).

Heterogeneity of residual standard deviations (SD) and coefficients of variation (CV) among effect classes was evaluated for each dataset in Table 1. Weaning weights were adjusted for effects of lamb age before analysis. Models included effects of flock and management group (nested within flock) and, for birth weight, effects of sex and sex x flock interaction. Models applied to each birth (for birth weights) or birth-rearing type (for weaning weights) also included effects of dam age class and dam age class × flock interaction. Models applied to each dam age class also included effects of birth (or birth-rearing) type and birth (or birth-rearing) type × flock interaction. Differences in residual variances among classes were assessed using F-ratios to variances in reference classes with Bonferroni adjustment for multiple comparisons as α/k where α is the target significance level and k is the number of comparisons [7].

Heterogeneity of residual SD and CV among flocks was assessed using subsets of records from core datasets in Table 1 that included only lambs from adult dams that were born as twins and, for weaning weights, also reared as twins. Records were analyzed separately for each flock using a model that included effects of management group and lamb sex for birth weights and management group (which included effects of lamb sex) for weaning weights. Means and residual SD and CV were used to quantify relationships between means and measures of variation among flocks.

### Comparison and validation of weaning weight adjustment procedures

In order to assess the impact of alternative adjustment procedures, weaning weight records of single lambs from seven flocks with the highest and seven flocks with the lowest mean weaning weights in the core dataset were corrected for effects of lamb age at weaning and adjusted for effects of birth-rearing type and dam age class using simple additive, simple multiplicative, or flock-specific multiplicative factors as described below. Flock-specific adjustments for TBR and dam age class were either made independently or included effects of TBR × dam age class interaction. Adjusted weaning weights from these divergently selected flocks were corrected for effects of management group (by absorption of effects of flock, birth year, and management group in the GLM Procedure of SAS®) and analyzed using a model that included effects of birth-rearing type and interactions of birth-rearing type with flock performance class and with flock nested within flock performance class. Linear contrasts were used to test differences between adjusted weaning weights of single and twin lambs within each flock performance class and the consistency of this difference between flock performance classes. The linear contrasts and the flock × performance class interaction were tested with the mean square of effects of birth-rearing type × flock within flock performance class interactions. For triplet lambs, the same procedure was applied using five high- and five low-performance flocks from the TBR = 33 dataset.

An additional comparison of alternative adjustment procedures for single lamb weights in an independent dataset was performed using records on 5012 twin and 2018 single lambs from 34 smaller flocks (150 to 499 weaning records per flock) that were not used in the original analyses. The numbers of observations in these flocks were considered too small to permit direct estimation of flock-specific adjustment factors, but these flocks represented a significant proportion of the overall NSIP dataset, and the behavior of alternative adjustment strategies in these smaller flocks was considered informative. Weaning weights from the nine flocks with the highest and the nine flocks with the lowest mean weaning weights were therefore adjusted using simple additive, simple multiplicative, or flock-specific multiplicative factors and differences between adjusted weaning weights of single and twin lambs were compared within and between groups of high- and low-performance flocks. Adjustment protocols for triplet lambs could not be evaluated in the validation dataset because the numbers of lambs with TBR = 33 (average of 20 per flock) were small.

## Results

### Birth weights

Birth weights in all datasets were influenced (*P* < 0.001) by effects of flock, birth type, dam age class, lamb sex, and dam age class × birth type interaction. Both additive and multiplicative adjustments that were required to correct birth weights of single lambs to a twin-lamb equivalent were larger for yearling and 2-year-old dams than for adult dams but did not differ greatly between adult and aged dams (Table 2). The factors that were required to adjust records of triplet lambs to a twin-lamb basis were similar for 2-year-old and adult dams but tended to be larger for aged dams. In the core data, additive and multiplicative factors that were required to adjust birth weights of ram lambs to a ewe-lamb equivalent were equal to −0.34 kg and 0.941, respectively.

**Table 2:** Least-squares (LS) means (kg) and adjustment factors for effects of birth type, dam age class, and their interaction on lamb birth weights

Data set	Dam age class, years	LS means for birth type				Additive adjustment (kg) for birth type		Multiplicative adjustment for birth type	
		1	2	3	Average	1	3	1	3
Core	2	6.31 ± 0.04	5.22 ± 0.03	4.44 ± 0.05	5.33 ± 0.03	−1.09	0.78	0.83	1.18
	3 to 6	6.61 ± 0.03	5.71 ± 0.03	4.90 ± 0.03	5.74 ± 0.02	−0.90	0.81	0.86	1.17
Yearling^a^	1	5.67 ± 0.04	4.53 ± 0.04	-	5.10 ± 0.04^c^	−1.14	-	0.80	-
	3 to 6	6.59 ± 0.04	5.66 ± 0.03	-	6.06 ± 0.03^c^	−0.91	-	0.86	-
Aged dams^b^	3 to 6	6.51 ± 0.03	5.64 ± 0.03	4.83 ± 0.03	5.66 ± 0.03	−0.87	0.81	0.87	1.17
	>6	6.48 ± 0.06	5.56 ± 0.04	4.68 ± 0.06	5.57 ± 0.04	−0.92	0.88	0.86	1.19

^a^Includes flocks from the core dataset that also have at least 50 records on yearling dams

^b^includes flocks from the core dataset that also have at least 50 records on aged dams

^c^excludes triplet lambs

**Fig. 1:**
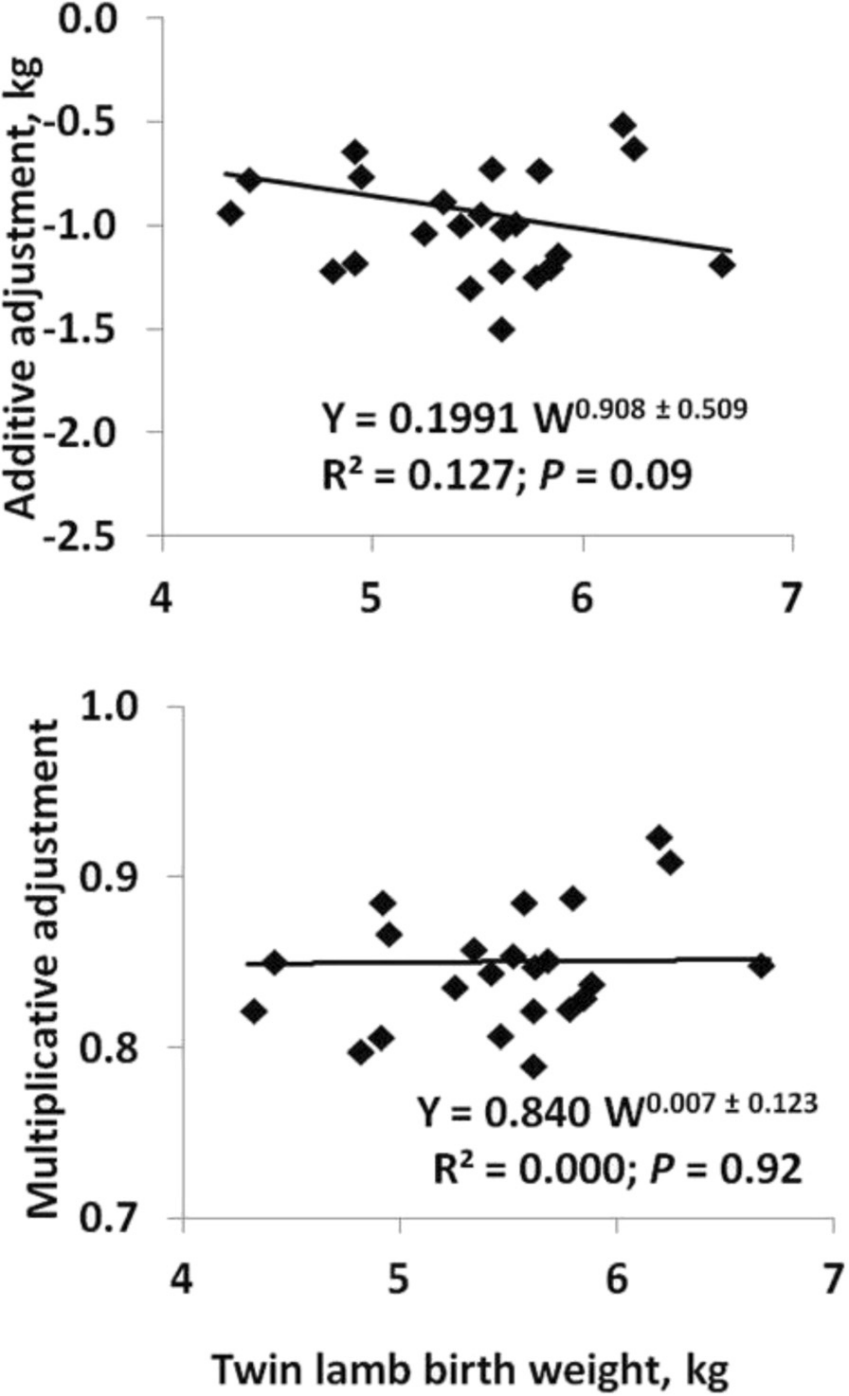
Relationship between adjustment factors and flock means for birth weight. Additive or multiplicative factors (Y) that are required to adjust birth weights of single lambs to a twin-lamb basis are plotted against flock means for birth weights of twin lambs (W). Prediction equations were derived by weighting each observation by the number of single-born lambs in the flock

Flock means for birth weights of twin lambs in the core data ranged from 4.3 to 6.7 kg (Fig. 1). Effects of birth type differed among flocks (*P* < 0.001) in all datasets. Effects of flock × dam age class interaction were likewise significant in all datasets (*P* ≤ 0.04) and most significant (*P* < 0.001) in the dataset that included yearling dams. Effects of flock × lamb sex interaction were significant (*P* ≤ 0.01) in core and aged-dam datasets and approached significance (*P* = 0.08) in the dataset that included yearling dams. In general, the additive factors that were necessary to adjust for effects of birth type were proportional to flock means. For example (Fig. 1), differences in birth weights between single- and twin-born lambs were proportional to the 0.91 ± 0.51 power of the flock mean for twin-lamb birth weight, whereas multiplicative factors that were necessary to adjust birth weights of single lambs to a twin-lamb equivalent were independent of flock means for birth weight (*P* = 0.92). Differences in birth weights between twin and triplet lambs (not shown) were likewise approximately proportional (1.15 ± 0.33) to flock means for twin-lamb birth weights whereas corresponding multiplicative factors were independent of flock means (*P* = 0.56). A somewhat different pattern (not shown) was observed for lambs from yearling dams: differences in birth weights between yearling and adult dams were proportional to the 2.12 ± 0.74 power of the flock mean for birth weights of lambs from adult dams. The corresponding proportionality constant for multiplicative factors was positive (0.26 ± 0.16) but not significantly different from zero (*P* = 0.13). However, the smaller additive and multiplicative factors that were required to adjust birth weights of lambs from 2-year-old or aged dams to an adult-dam equivalent or to adjust weights of ram lambs to a ewe-lamb basis (not shown) were not associated with flock means for birth weight.

### Weaning weights

Weaning weights in all datasets were influenced (*P* < 0.001) by effects of flock, birth-rearing type, and dam age class. In general, effects of birth-rearing type × dam age class interaction were also significant (*P* ≤ 0.04). Both additive and multiplicative adjustments to correct weights of lambs born and reared as single lambs to a twin-lamb basis were larger for yearling, 2-year-old, and aged dams than for adult dams (Table 3), but, in general, differences among additive factors were only marginally significant (e.g., *P* = 0.05 for 2-year-old versus adult dams) and differences among dam age classes in multiplicative factors were at most equal to 0.04. No meaningful differences in adjustment factors between 2-year-old and adult dams were observed for the less-numerous triplet lambs (not shown). The average multiplicative factor that was necessary to adjust weaning weights of lambs with TBR = 33 to a twin birth-rearing type equivalent was equal to 1.14.

**Table 3:** Least-squares (LS) means (kg) and adjustment factors for effects of birth-rearing type, dam age class, and their interaction on lamb weaning weights of single and twin lambs

Data set	Dam age class	LS means for birth-rearing type			Single lamb adjustment factor	
		11	22	Average	Additive, kg	Multiplicative
Core	2	31.4 ± 0.2	26.3 ± 0.1	28.8 ± 0.1	−5.1	0.84
	3 to 6	32.6 ± 0.2	28.1 ± 0.1	30.4 ± 0.1	−4.5	0.86
Yearling^a^	1	29.1 ± 0.2	24.1 ± 0.2	26.6 ± 0.2	−4.9	0.83
	3 to 6	32.9 ± 0.2	28.5 ± 0.2	30.7 ± 0.2	−4.4	0.87
Aged dams^b^	3 to 6	29.8 ± 0.2	25.4 ± 0.2	27.7 ± 0.2	−4.5	0.85
	>6	28.8 ± 0.3	24.1 ± 0.2	26.5 ± 0.2	−4.7	0.84

^a^Includes flocks from the core dataset that also have at least 50 records on yearling dams

^b^includes flocks from the core dataset that also have at least 50 records on > 6-year-old dams

**Fig. 2:**
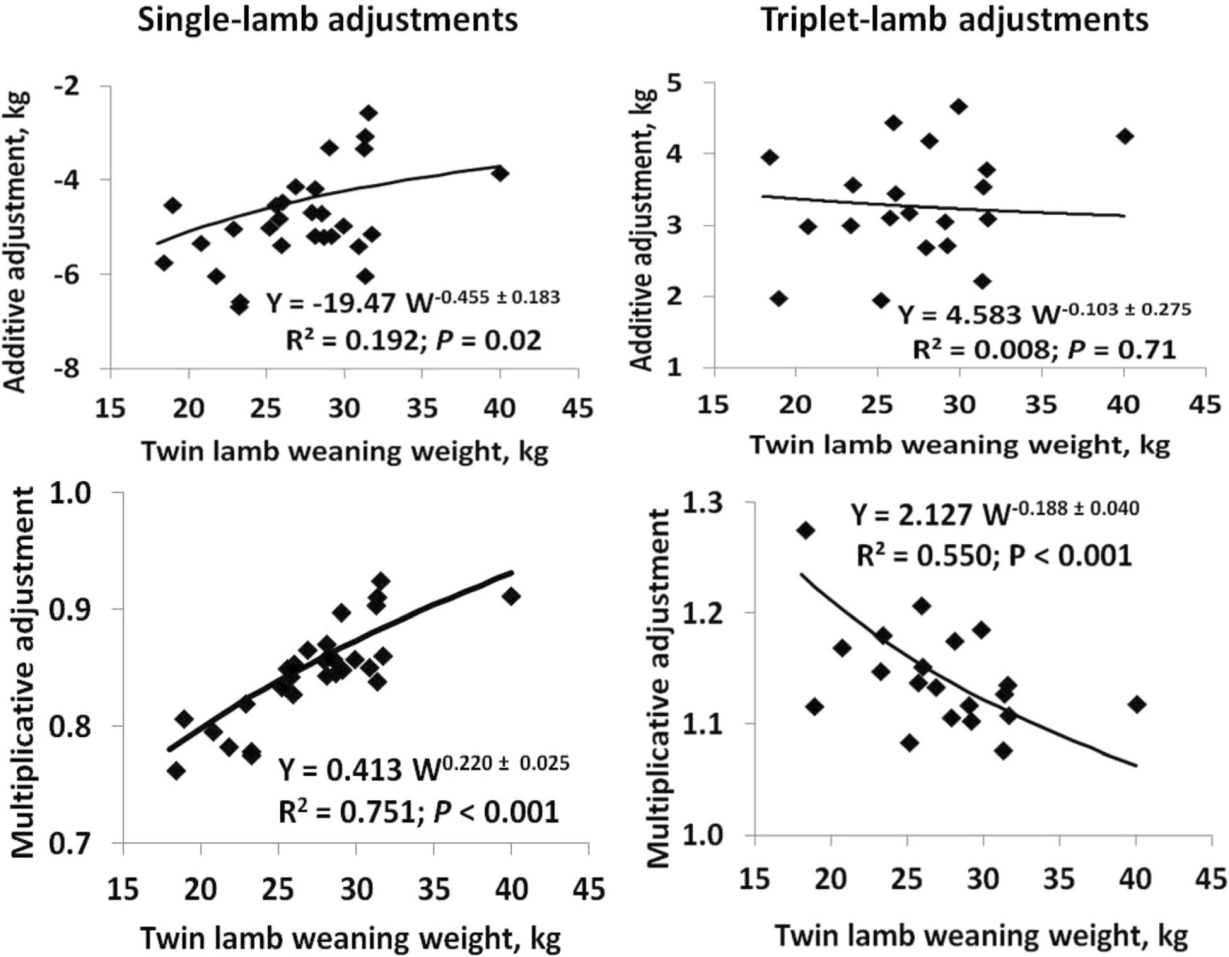
Relationships between adjustment factors and flock means for weaning weight. Additive or multiplicative factors (Y) that are required to adjust weaning weights of lambs born and reared as single individuals or triplets to a twin-born, twin-reared lamb basis are plotted against flock means for weaning weights of twin-born, twin-reared lambs. Prediction equations for single-born, single-reared lambs were derived by weighting each observation by the number of single-born, single-reared lambs in the flock. Prediction equations for triplet-born, triplet-reared lambs were derived by weighting each observation by the number of triplet-born, triplet-reared lambs in the flock

Effects of birth-rearing type varied among flocks (*P* < 0.001) in all datasets (Fig. 2). Differences in weaning weights between single and twin lambs became smaller as flock means for twin lambs increased (*P* = 0.02), with a corresponding positive relationship (*P* < 0.001) between multiplicative adjustment factors and flock means. However, this relationship was much stronger for multiplicative factors than for additive factors, with coefficients of determination (R^2^) of 0.75 and 0.19, respectively. For triplet lambs, both additive and multiplicative adjustment factors were inversely related to flock means for weaning weights of twin lambs (Fig. 2), which again indicated smaller effects on weaning weights for triplet lambs in high-performance flocks. R^2^ values were again much larger for multiplicative than for additive factors (0.55 and 0.01, respectively). Thus, application of a single set of multiplicative factors to adjust weaning weights of single and triplet lambs to a twin-lamb basis across a wide range of flock performance levels resulted in biased adjustments in some flocks. Multiplicative factors for single and triplet weaning weights were proportional to the 0.22 ± 0.02 and −0.19 ± 0.04 powers, respectively, of flock means for weaning weights of twin lambs. As flock means for twin lambs from adult ewes increased from 18 to 40 kg, predicted multiplicative adjustment factors for single lambs increased from 0.78 to 0.93 and those for triplet lambs declined from 1.24 to 1.06.

Many ewes that produced triplets did not suckle all their lambs, which was due either to lamb death losses or a management decision to reduce the size of the litter by artificial rearing or fostering of one or more lambs to another ewe. Selection of triplet-bearing ewes that were allowed to nurse all of their lambs may impact the realized performance of ewes nursing triplets. The average percentage of triplet-born lambs that were reared as triplets (T) was equal to 47 % and varied from 17 to 81 % for the 20 flocks in Fig. 2. Addition of T to the model that was used to derive the power function for triplets in Fig. 2 revealed a positive effect (*P* = 0.02) of T. The resulting prediction equation was Y = 1.91 W^-0.171^e^0.0010T^ and predicted that the multiplicative adjustment factor for triplet weaning weights at the mean flock performance level would increase from 1.10 to 1.17 as the proportion of triplet-born lambs that were reared as triplets increased from 20 to 80 %. Addition of T to the model had little effect on the relationship shown in Fig. 2 because the correlation of T with the flock mean for twin lambs was only −0.04 (*P* = 0.86), but R^2^ increased from 0.55 to 0.68. Thus, the proportion of triplet-bearing ewes that actually nursed all of their lambs influenced the average performance of those lambs relative to their twin-born contemporaries.

Effects of flock × birth-rearing type interactions were significant in datasets that included lambs with TBR = 32 or 21 (*P* < 0.001). The relationship between resulting flock-specific multiplicative adjustment factors and flock means for weaning weights of twin lambs was significant for TBR = 21 (*P* = 0.04; β = 0.10 ± 0.04; average multiplicative adjustment = 0.94) but not for TBR = 32 (*P* = 0.42; β = −0.03 ± 0.04; average multiplicative adjustment = 1.06). Thus, the results for these low-frequency birth-rearing types were broadly consistent with those for TBR = 22 and 33, but added little new information and will not be discussed further.

Effects of flock × dam age class interaction were significant (*P* ≤ 0.02) in all datasets and most significant (*P* = 0.001) in the data that included yearling dams. Neither additive (*P* ≥ 0.36) nor multiplicative (*P* ≥ 0.10) factors to adjust lamb weaning weights to an adult-ewe basis were associated with flock means for weaning weight, although the multiplicative factor for yearling dams approached significance (*P* = 0.10 and R^2^ = 0.19 with a proportionality constant of −0.11; not shown). In yearling dams, multiplicative adjustment factors were predicted to decline from 1.19 to 1.13 as flock mean weaning weights increased from 21 to 35 kg.

### Development of adjustment protocols

Differences in adjustment factors for birth-rearing type and dam age class between flocks were quite repeatable in our data, as documented by significant effects of flock × birth-rearing type and flock × dam age class interactions when these effects were tested against effects of flock × birth year × birth-rearing type and flock × birth year × dam age class interactions, respectively. A relationship generally existed between multiplicative adjustment factors for these effects and flock means for weaning weight, although these relationships were large and clearly significant only for the factors that were required to adjust weaning weights of single and triplet lambs to a twin-lamb basis. These results suggested that flock-specific adjustment factors could be derived using means for body weights of animals in reference classes. If **F** is an array that contains multiplicative adjustment factors F_ij_ for the i^th^ dam age class and j^th^ birth-rearing type in the population as a whole and $\overline{W}$ is the population mean weight for lambs in the reference class (i.e., twin-born, twin-reared lambs from adult dams), then, in the absence of birth-rearing type x dam age class interaction, adjustment factors for flock k can be derived as:

1 $$ {\tilde{F}}_{ijk}={\tilde{F}}_{ik}{\tilde{F}}_{jk}={\tilde{F}}_i{\left(\frac{{\overline{W}}_k}{\overline{W}}\right)}^{\beta_i}{F}_j{\left(\frac{{\overline{W}}_k}{\overline{W}}\right)}^{\beta_j}={F}_i{F}_j{\left(\frac{{\overline{W}}_k}{\overline{W}}\right)}^{\beta_i+{\beta}_j} $$

where β_i_ and β_j_ are coefficients of power function for birth-rearing types and dam age classes, respectively, and ${\overline{W}}_k$ is the mean weight of lambs in the reference class for flock k. For example, the adjusted weaning weight for a single lamb m with an adult dam in the k^th^ flock would be as follows:

$$ {\tilde{W}}_{ijkm}=0.85{W}_{ijkm}{\left(\frac{{\overline{W}}_k}{\overline{W}}\right)}^{0.222}, $$

where 0.85 is the simple multiplicative adjustment factor for single lambs and 0.222 is the power function from Fig. 2 used to derive flock-specific multiplicative adjustment factors for single lambs. Similarly, the adjusted weaning weight for a single lamb from a yearling dam in the k^th^ flock would be as follows:

$$ {\tilde{W}}_{ijkm}=0.85(1.16){W}_{ijkm}{\left(\frac{{\overline{W}}_k}{\overline{W}}\right)}^{0.222-0.105}. $$

To incorporate dam age class × birth-rearing type interaction in a flock-specific adjustment protocol, it was necessary to model the joint effects of these variables on adjustment factors. In the presence of dam age class × birth-rearing type interaction, $$ {\tilde {F}}_{ij} $$ ≠ $$ {\tilde {F}}_i $$$$ {\tilde {F}}_j $$ and adjustment requires the estimation of $$ {\tilde {F}}_{ij} $$ and β_ij_ for each subclass, which is difficult because the numbers of observations are smaller in subclasses than in larger main effect classes. However, if effects of interactions on $$ {\tilde {F}}_{ij} $$ reflect underlying effects of interactions on F_ij_ in Equation (1), but approximately independent effects on scalers β_i_ and β_j_, then flock-specific adjustment factors become:

2 $$ {\tilde{F}}_{ijk}={F}_{ij}{\left(\frac{{\overline{W}}_k}{\overline{W}}\right)}^{\beta_i+{\beta}_j}. $$

Multiplicative adjustment factors for weaning weights that incorporated effects of birth-rearing type × dam age class interaction were derived by adding records with TBR = 33 and records for yearling and aged dams to the core dataset in Table 1 and fitting effects of all TBR × dam age class subclasses (Table 4). However, the number of observations with TBR = 33 was small for yearling dams (n = 18) and the multiplicative adjustment factor derived from the data was smaller than expected (1.17); this is presumably linked to the small numbers of observations and the selection of yearling dams that were allowed to rear all three of their lambs. Multiplicative adjustment factors for lambs with TBR = 33 and raised by aged dams were based on larger numbers of records (n = 168) and not obviously inappropriate relative to those obtained for other dam age classes (Table 4), but they may have been liable to similar, but smaller, biases that are analogous to those hypothesized for yearling dams. Thus, an alternative adjustment factor was required for yearling dams with TBR = 33 and was derived by multiplying the corresponding factor for 2-year-old dams by the ratio of adjustment factors for 1- and 2-year-old ewes raising twin lambs (e.g., as 1.22 × (1.18/1.07) = 1.35). We hypothesized that this derived adjustment factor was more representative of the actual class effect associated with unselected yearling dams.

**Table 4:** Relative frequencies and additive and multiplicative adjustment factors to correct weaning weights for the interaction of birth-rearing type (TBR) and dam age class^a^

Type of factor	TBR	Dam age class, years				
		1	2	3 to 6	>6	Average
Frequency, %	11	5.5	6.9	12.4	2.2	27.0
	22	4.5	16.4	38.9	5.0	64.8
	33	0.1	1.1	6.4	0.6	8.2
	Sum	10.1	24.4	57.7	7.8	100.0
Additive, kg	11	−0.55	−3.26	−4.49	−3.36	−4.76
	22	4.38	1.87	0.00	1.31	0.00
	33	7.77^b^	5.26	3.39	4.70	3.39
	Average	4.11	1.63	0.00	1.28	
Multiplicative	11	0.98	0.90	0.86	0.88	0.85
	22	1.18	1.07	1.00	1.05	1.00
	33	1.35^b^	1.22	1.14	1.20	1.14
	Average	1.16	1.06	1.00	1.05	

^a^Factors were derived from least-squares means for TBR × dam age class subclasses

^b^adjustment factors for this class could not be accurately estimated from the available data due to low subclass frequencies. Thus, additive adjustment factors for yearling dams with TBR = 33 were derived by adding corresponding additive factors for 2-year-old dams to the difference between 1- and 2-year-old ewes that raise twin lambs (e.g., as 5.26 + (4.38 -1.87 = 7.77). Multiplicative adjustment factors for these lambs were derived by multiplying corresponding factors for 2-year-old dams by the ratio of adjustment factors for 1- and 2-year-old ewes that raise twin lambs (e.g., as 1.22 × (1.18 / 1.07) = 1.35)

### Magnitude of adjustment bias in high- and low-performance flocks in core and triplet data

The impact of alternative adjustment protocols on adjusted weaning weights in high- versus low-performance flocks was substantial for both single and triplet lambs and also significant for lambs from yearling dams. The mean difference in adjusted weaning weight between single and twin lambs in low-performance flocks was equal to 0.72 ± 0.48 kg (*P* = 0.16) following the application of a simple additive adjustment factor but reached 1.26 ± 0.42 kg (*P* = 0.01) if a simple multiplicative factor was applied (Table 5). In high-performance flocks, comparable differences were equal to −0.82 ± 0.55 kg (*P* = 0.16) and −1.89 ± 0.48 kg (*P* = 0.002) after application of simple additive or multiplicative adjustments, respectively. Thus, the effect of flock performance level on differences in apparent bias in adjusted weaning weights for single lambs in high- versus low-performance flocks was highly significant for the simple multiplicative adjustment (*P* < 0.001) and approached significance (*P* = 0.06) for the simple additive adjustment. Use of flock-specific multiplicative adjustment factors (Fig. 2) reduced differences between single lambs and twins in adjusted weaning weights to 0.19 ± 0.43 kg (*P* = 0.66) and −0.32 ± 0.49 kg (*P* = 0.53) in low- and high-performance flocks, respectively. These differences did not differ between low- and high-performance flocks (*P* = 0.45). Addition of effects of birth-rearing type × dam age class interaction to the flock-specific multiplicative adjustments did not reduce the apparent prediction bias within flock performance levels but increased consistency of predictions between high- and low-performance flocks.

**Table 5:** Differences between adjusted weaning weights of single-born, single-reared and twin-born, twin-reared lambs (expressed as single - twin) in high- versus low-performance flocks from the core and validation data sets following application of different adjustment protocols

Data set	Adjustment protocol	Flock performance level		Difference (low – high)
		Low	High	
Core	Simple additive	0.72 ± 0.48	−0.82 ± 0.55	1.54 ± 0.73^†^
	Simple multiplicative	1.26 ± 0.42^**^	−1.89 ± 0.48^**^	3.16 ± 0.64^***^
	Flock-specific multiplicative	0.19 ± 0.43	−0.32 ± 0.49	0.51 ± 0.65
	Simple multiplicative with interaction	1.70 ± 0.42^**^	−1.29 ± 0.48^*^	2.98 ± 0.64^***^
	Flock-specific multiplicative with interaction	0.62 ± 0.44	0.32 ± 0.50	0.31 ± 0.67
Validation	Simple additive	−0.55 ± 0.80	−1.89 ± 0.54^**^	1.34 ± 0.97
	Simple multiplicative	−0.35 ± 0.68	−2.56 ± 0.46^***^	2.21 ± 0.82^*^
	Flock-specific multiplicative	−0.83 ± 0.69	−1.44 ± 0.47^**^	0.61 ± 0.84
	Simple multiplicative with interaction	0.11 ± 0.68	−1.96 ± 0.46^***^	2.07 ± 0.82^*^
	Flock-specific multiplicative with interaction	−0.38 ± 0.71	−0.88 ± 0.48^†^	0.51 ± 0.86

Based on seven (of 28) flocks in the core dataset (Table 1) and nine (of 34) flocks in validation data with the lowest flock means for weaning weight and on seven flocks in the core dataset and nine flocks in the validation data with the highest flock means for weaning weight; ^†^, *, **, ***: *P* < 0.10, 0.05, 0.01, and 0.001, respectively

Comparisons of adjusted weaning weights for twin and triplet lambs in the triplet dataset in Table 1 yielded similar results (Table 6) to those observed for single and twin lambs. A simple multiplicative adjustment factor led to differences in adjusted weaning weights between twins and triplets in low- and high-performance flocks of 0.97 ± 0.52 kg (*P* = 0.10) and −0.85 ± 0.55 kg (*P* = 0.16), respectively, and the effect of flock performance level on apparent adjustment bias was significant (*P* = 0.04). However, use of simple additive adjustment factors resulted in no apparent systematic adjustment bias (*P* = 0.76; Table 6) and flock-specific multiplicative adjustments, either with or without consideration of effects of birth-rearing type × dam age class interaction, were not superior to simple additive adjustment factors in level of apparent bias (*P* = 0.48) for less-numerous triplet lambs.

**Table 6:** Differences between adjusted weaning weights of twin-born, twin-reared and triplet-born, triplet-reared lambs (expressed as twin – triplet) in high- versus low-performance flocks from the TBR = 33 dataset by applying different adjustment protocols

Adjustment protocol	Flock performance level		Difference (low – high)
	Low	High	
Simple additive	0.18 ± 0.55	0.03 ± 0.58	0.15 ± 0.80
Simple multiplicative	0.97 ± 0.52^†^	−0.85 ± 0.55	1.82 ± 0.76^*^
Flock-specific multiplicative	−0.03 ± 0.69	0.71 ± 0.73	−0.74 ± 1.00
Simple multiplicative with interaction	0.97 ± 0.52	−0.84 ± 0.55	1.81 ± 0.76^*^
Flock-specific multiplicative with interaction	−0.03 ± 0.69	0.71 ± 0.73	−0.74 ± 1.00

Based on five (of 20) flocks with the lowest flock means and five flocks with the highest flock means for weaning weight in the TBR = 33 dataset (Table 1); ^†^, *: *P* < 0.10 and 0.05, respectively

### Magnitude of adjustment bias in high- and low-performance flocks in validation data

Differences in adjusted weights between single and twin lambs in validation data were generally underestimated in both high- and low-performance flocks (Table 5), but the apparent bias was not significant in low-performance flocks (*P* ≥ 0.25). By contrast, and in agreement with results from core data, adjusted weights of single lambs appeared to be biased downwards in high-performance flocks. The extent of this bias was largest when simple multiplicative adjustment factors were applied, either with or without consideration of effects of birth-rearing type x dam age class interaction (*P* < 0.001); it was reduced but remained significant if either simple additive or flock-specific multiplicative factors (*P* = 0.003 and *P* =0.007, respectively) were used; and it was removed (*P* = 0.09) only if both effects of interaction and scaling of multiplicative factors for differences in flock means were considered.

### Heterogeneity of variances

**Fig. 3:**
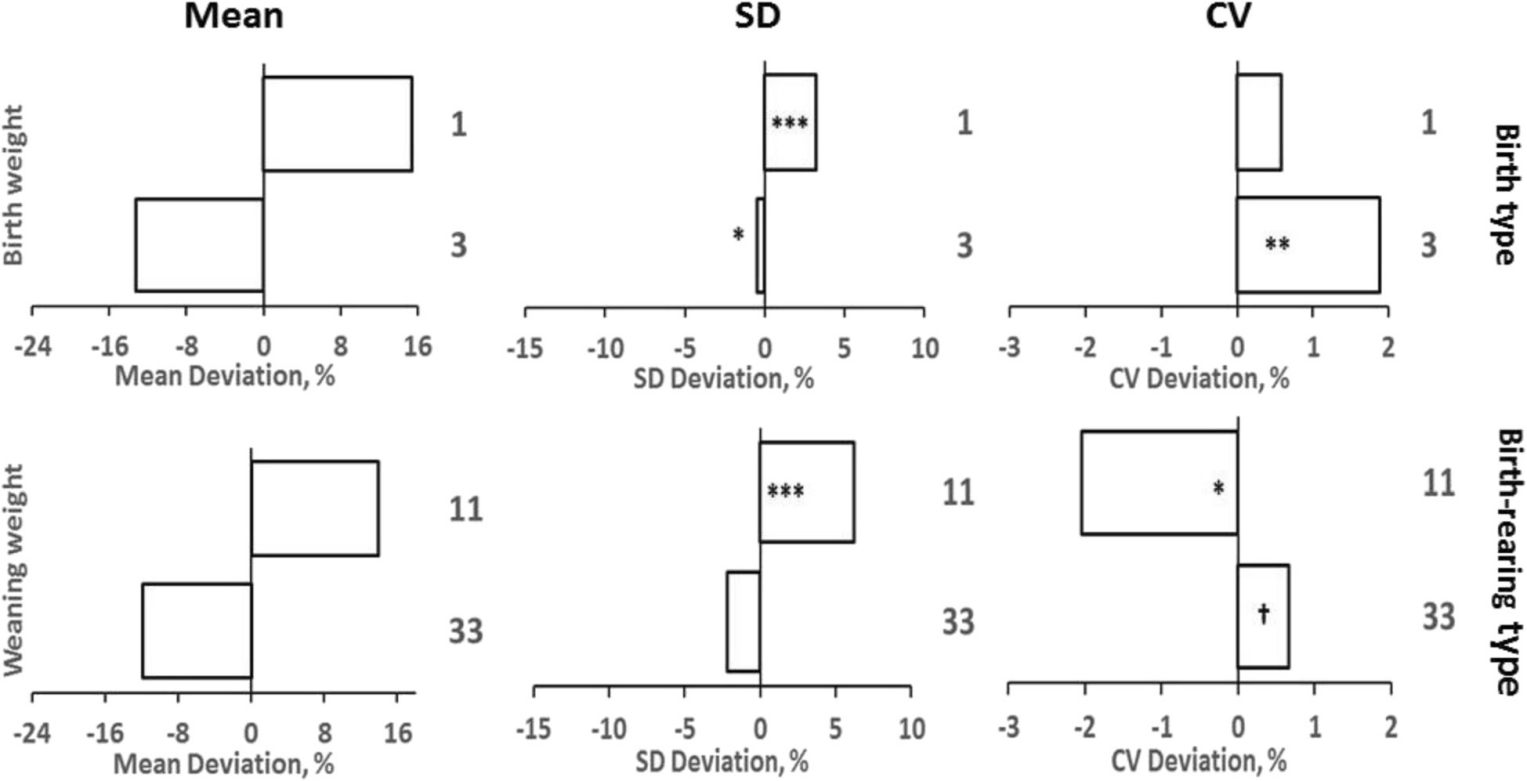
Means and residual standard deviations and coefficients of variation among birth types for birth weights and among birth-rearing types for weaning weights. Means and standard deviations (SD) are expressed as percentage deviations from the reference class (twin-born lambs for birth weight and twin-born, twin-reared lambs for weaning weights). Coefficients of variation (CV) are expressed as absolute deviations from the reference class. ^†^, *, **, *** indicate that residual variances or CV differ from those observed for twin lambs at *P* < 0.10, 0.05, 0.01, and 0.001, respectively, by F-test with Bonferroni adjustment for multiple comparisons

**Fig. 4:**
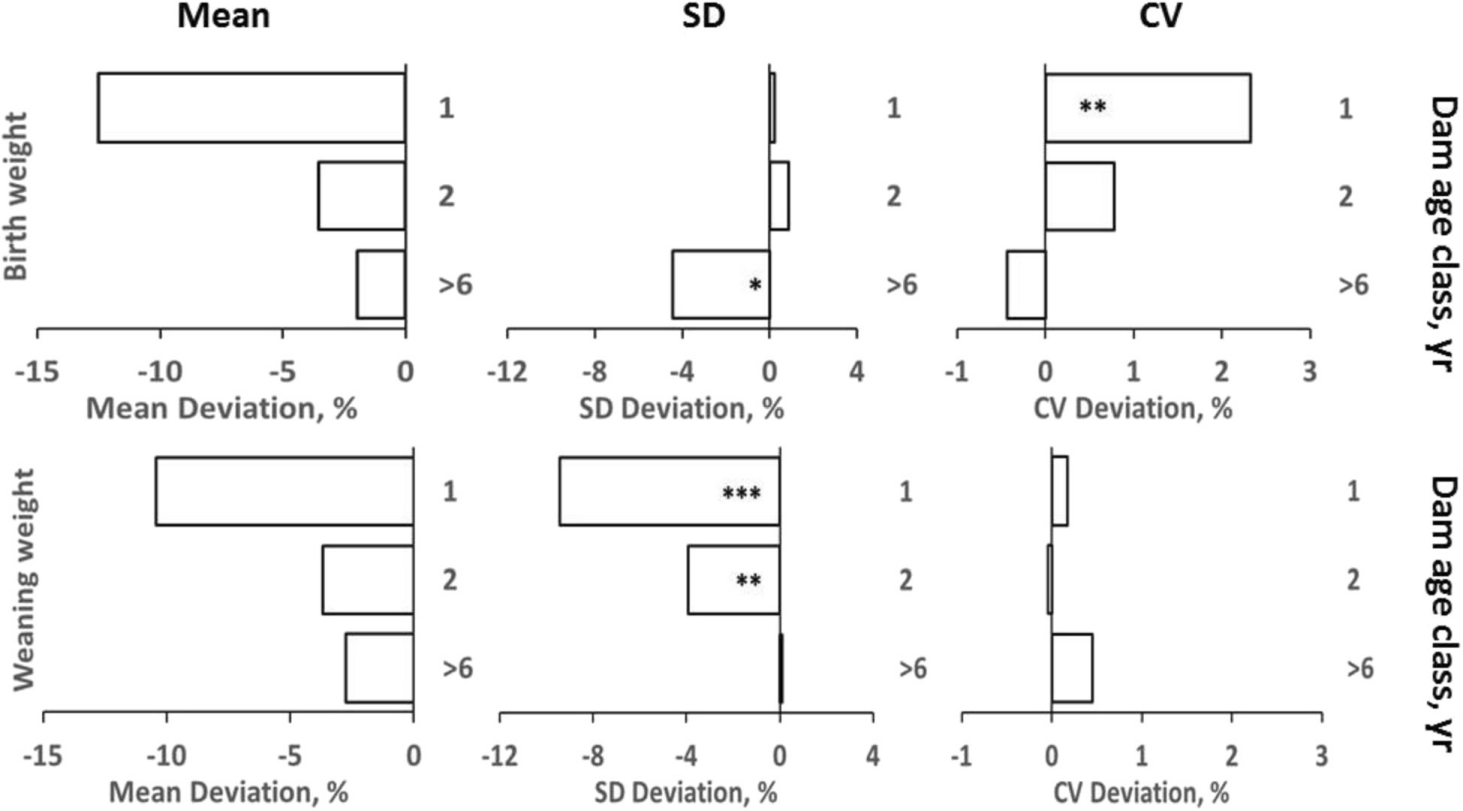
Means and residual standard deviations and coefficients of variation among dam age classes for birth and weaning weights. Means and standard deviations (SD) are expressed as percentage deviations from the reference class (3- to 6-year-old dams). Coefficients of variation (CV) are expressed as absolute deviations from the reference class. *, **, *** indicate that residual variances or CV differ from those observed for 4- to 6-year-old dams at *P* < 0.05, 0.01, and 0.001, respectively, by F-test with Bonferroni adjustment for multiple comparisons

A degree of proportionality between means and SD was observed for most effect classes (Figs. 3 and 4), but SD for birth weights did not differ among lambs with yearling, 2-year-old, and adult dams. Birth weight CV for triplet lambs and lambs from yearling dams were higher (*P* < 0.001) than those for lambs in reference classes. For weaning weights, CV among dam age classes were within a 1 % range of CV for adult dams, which suggests that multiplicative adjustments would approximately equalize residual variances among dam age classes. However, the weaning weight CV for single lambs was lower than those observed for twin or triplet lambs.

**Fig. 5:**
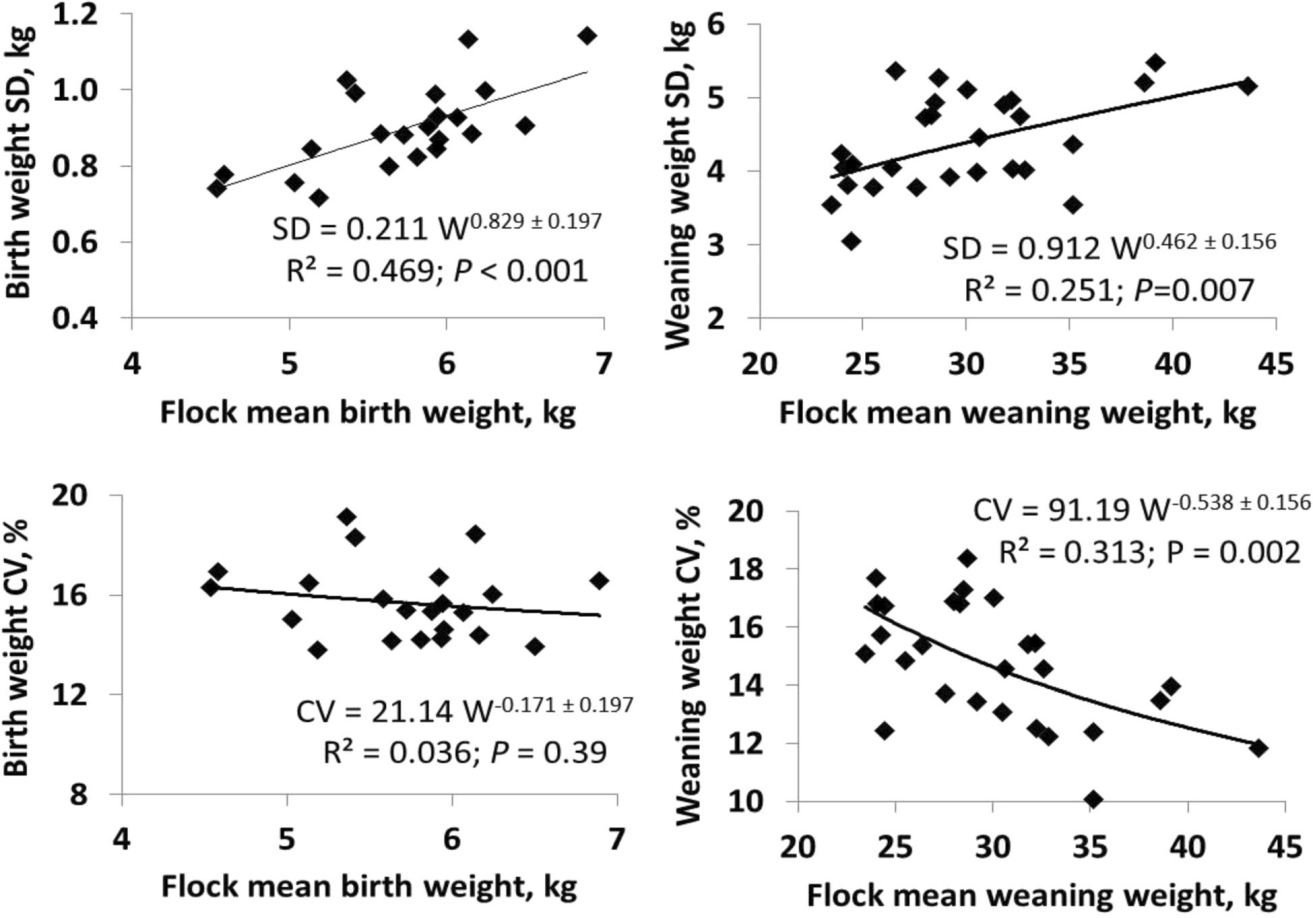
Flock differences in residual standard deviations and coefficients of variation. Standard deviations (SD) and coefficients of variation (CV) are for birth weights of twin-lambs from 3- to 6-year-old dams and weaning weights of twin-born, twin-reared lambs from 3- to 6-year-old dams. Power functions describe associations of residual SD and CV to flock means

Residual SD for weights of twin lambs from 3- to 6-year-old dams were positively associated with flock means (*P* < 0.01; Fig. 5). The proportionality constant (β) that relates SD to flock means did not differ from 1.0 for birth weights (β = 0.83 ± 0.26) but was proportional to only the 0.46 ± 0.16 power of flock means for weaning weights, with a corresponding negative association between means and residual CV (β = −0.54 ± 0.16; *P* = 0.002). Residual SD among flocks were approximately equalized for birth weights by scaling observations by flock means, but scaling by approximately the square root of flock means was required to equalize SD for weaning weights.

## Discussion

The birth-rearing type of the lamb has a major influence on pre-weaning growth, and adjustment of phenotypic records for effects of birth-rearing type is essential for the genetic evaluation of body weights in sheep. Use of multiplicative factors to accomplish this adjustment is widespread [8, 9] and assumes that differences among birth-rearing types are proportional to the mean level of performance. However, this study documented flock differences in effects of birth-rearing type on lamb weaning weights, with an inverse relationship between flock means for weaning weight and multiplicative effects of birth-rearing type. Thus, the use of a common set of multiplicative adjustment factors across flocks that differ widely in mean weaning weight resulted in under-adjustment of weights for lambs born and reared as twins or triplets in low-performing flocks and over-adjustment of weights of similar lambs in high-performance flocks. Estimated breeding values for single lambs would thus be biased upward in low-growth flocks and downward in high-growth flocks. Effects of flock mean performance on adjustment factors for dam age were smaller than those observed for birth-rearing type, but approached significance in datasets that included records from yearling dams, with an inverse relationship between flock means for weaning weight and multiplicative factors to adjust records of lambs from yearling dams to an adult-ewe basis. By contrast, simple multiplicative adjustments for birth weights appeared adequate to account for flock differences in effects of both birth type and dam age.

Potential to derive flock-specific adjustment factors has been acknowledged [10, 11] but rarely implemented in genetic evaluation systems. Industry datasets often include many flocks that do not have adequate numbers of records to permit straightforward development of flock-specific adjustment factors. There is also some concern regarding the repeatability of flock-specific factors across years and management groups and potential for flock-specific factors to change over time in association with changes in environmental conditions or flock management. Our results indicate that, in flocks with a minimum of 500 weight records, differences in adjustment factors for birth-rearing type and dam age class between flocks were relatively repeatable across years, with highly significant effects of flock × birth-rearing type and flock × dam age class interactions when those effects were tested against effects of random flock × birth-rearing class × birth year and flock × dam age class × birth year interactions. This observation does not, however, preclude changes in management, and therefore in the optimum array of adjustment factors, for individual flocks.

Several studies in the 1970s and 1980s addressed the adjustment of body weights of calves and lambs for effects of sex, dam age, and, for lambs, type of birth and rearing. Comparisons of additive versus multiplicative adjustment factors generally focused on achieving homogeneity of variances among effect classes. For effects of type of birth and rearing in sheep, proportionality between means and variances was often observed, with similar CV among classes [12]. These results supported the use of multiplicative factors to standardize residual SD of weaning and post-weaning weights among birth-rearing types. In both cattle and sheep, additive factors were generally recommended to adjust for effects of dam age. For single- and twin-born Rambouillet lambs with 2- to 6-year-old dams at a single location, 120-d weaning weights of lambs from younger dams were smaller but more variable than those from adult (4-year-old) dams [12]. In beef cattle, the US Beef Improvement Federation recommended the use of additive factors to adjust for effects of dam age [13], which reflects results reported in [14] that showed a greater variation relative to the mean in progeny of younger dams.

Heterogeneity of variances among effect classes allows individuals that are in more variable classes to have a greater impact on resulting EBV. Thus, the use of additive adjustment factors would be expected to result in larger weaning weight deviations from management-group means for single lambs and smaller deviations for lambs with 1- and 2-year-old dams. By contrast, CV for the various effect classes indicated that multiplicative adjustment factors tended to equalize variances among classes and, therefore, more nearly equalized impacts on EBV of weaning weight records from different types of lambs, but would overcompensate for the larger SD observed in single lambs. Thus, our results agree with previous studies that indicated that accounting completely for both heterogeneity of variance among effect classes and flock differences in adjustment factors would require relatively complex adjustment protocols [9, 15].

Interactions among non-genetic effects can also influence adjustment protocols. In sheep, analyses of data from breeds and flocks with reasonable incidences of triplet births and records from yearling dams generally revealed significant effects of dam age × birth-rearing type interactions [1, 10], with larger multiplicative and much larger additive adjustment factors required to correct records from twin and, especially, triplet lambs to a single-lamb basis for lambs from younger and older dams. However, effects of dam age × birth-rearing type interaction for weaning weights were not observed in data that did not contain triplet lambs or yearling dams [12].

In the current study, flock differences in multiplicative factors (F) to adjust weaning weights for effects of birth-rearing type and dam age class were strongly associated with flock differences in mean weights (W) of lambs in reference classes (i.e., lambs with TBR = 22 and raised by 3- to 6-year-old dams) and could be modeled using prediction equations of the form F = αW^β^. These relationships allowed the prediction of flock-specific adjustment factors to be extended to larger numbers of flocks, because derivation of flock-specific factors required only an estimate of the mean weights of lambs in the reference class for each flock. These results conflict, in part, with those observed in 12 large New Zealand Romney flocks [15, 16]. In those flocks, significant variation was observed among flocks and years in adjustment factors for effects of lamb sex, birth-rearing type and dam age, but no association was reported between resulting factors and flock means for weaning weights. However, the magnitude of the range in means among those flocks was only 5.6 kg (21.4 to 27.0 kg), as compared to that of over 20 kg in our study. Given the diversity in weaning weight means in our data, we propose that accounting for effects of flock × birth-rearing type interaction properly is much more important than achieving full equality of residual SD among effect classes.

Development of adjustment protocols for weaning weights that can address the differences among flocks identified in this study requires that theoretical and operational considerations are balanced. Simple additive or multiplicative adjustment factors are easy to derive and apply, but our data suggest that simple multiplicative factors resulted in biased adjustments if differences in performance among flocks are large. Additive factors were somewhat less effective in equalizing variances among fixed effect classes, but more effective in accounting for across-flock bias. Flock-specific multiplicative factors based on the mean level of performance of the flock were equal or superior to simple additive factors, but required estimation of weaning weight means for animals in the reference class for each flock. The repeatability of flock effects in our analyses was generally high, but concern still exists regarding the potential for changes in flock means among years and management groups. Thus, a protocol that would extend the use of performance-based multiplicative factors to individual years or management groups becomes attractive. However, in our data, the average number of lambs in a weaning weight management group was 23, so accurate assessment of weaning weight means for twin lambs with adult dams was not possible for many management groups. It is possible to consider strategies that use mixed model or Bayesian approaches to predict weaning weight means for lambs in the reference classes in individual management groups as frequency-dependent combinations of overall, flock, and management group means. However, rapid updating is critical to effectively use EBV; LAMBPLAN EBV are normally updated every two weeks. Use of customized adjustments would require comparable frequent updating of adjustment factors and thus, it would probably be feasible only if factors can be derived as a simple function of weaning weight records.

## Conclusions

Important differences among flocks were observed in the adjustment factors that are required to correct weaning weights of single and triplet lambs to a twin-lamb equivalent. These differences were inversely related to flock means for weaning weights of twin lambs, which indicates that increases in flock performance resulted in smaller differences among birth-rearing types. Thus, the use of simple multiplicative adjustment factors led to substantial adjustment bias when applied across flocks with large differences in mean performance. This bias was reduced by using additive adjustment factors and could also be removed by using flock-specific multiplicative factors derived as simple functions of flock means for weaning weight.
